# Different Choices of Drinking Water Source and Different Health Risks in a Rural Population Living Near a Lead/Zinc Mine in Chenzhou City, Southern China

**DOI:** 10.3390/ijerph121114364

**Published:** 2015-11-12

**Authors:** Xiao Huang, Liping He, Jun Li, Fei Yang, Hongzhuan Tan

**Affiliations:** 1 Department of Epidemiology and Health Statistics, School of Public Health, Central South University, Changsha, Hunan 410008, China; E-Mail: huangxiao1998@163.com; 2 Department of Hygiene, School of Public Health, Xiangnan University, Chenzhou, Hunan 423000, China; E-Mail: lijun5006@126.com; 3 Department of Occupational Health and Environmental Health, School of Public Health, Central South University, Changsha, Hunan 410008, China; E-Mail: phfyang@csu.edu.cn

**Keywords:** drinking water, heavy metals, risk assessment, environmental epidemiology, mining activities

## Abstract

This study aimed to describe the households’ choices of drinking water sources, and evaluate the risk of human exposure to heavy metals via different drinking water sources in Chenzhou City of Hunan Province, Southern China. A cross-sectional face-to-face survey of 192 householders in MaTian and ZhuDui village was conducted. The concentrations of heavy metals in their drinking water sources were analyzed. Carcinogenic and non-carcinogenic risk assessment was performed according to the method recommended by the United States Environmental Protection Agency. In total, 52.60% of the households used hand-pressed well water, and 34.89% used barreled water for drinking. In total, 6.67% of the water samples exceeded the Chinese drinking water standards. The total health risk of five metals is 5.20 × 10^−9^~3.62 × 10^−5^. The total health risk of five metals was at acceptable levels for drinking water sources. However, the total risk of using hand-pressed well water’s highest value is 6961 times higher than the risk of using tap water. Household income level was significantly associated with drinking water choices. Arsenic (As) and lead (Pb) are priority controlled pollutants in this region. Using safe drinking water (tap water, barreled water and so on) can remarkably reduce the risk of ingesting heavy metals.

## 1. Introduction

Safe drinking water is universally recognized as critical components of public health. Heavy metal contamination, such as from lead (Pb), cadmium (Cd), mercury (Hg) and metalloid arsenic (As), in drinking water has been receiving increasing attention in China recently [1,2,3]. Health risk assessment is crucial to understanding the potential health risks from heavy metal exposure in humans. This information is very important for decision-makers when setting up policies or regulations to protect the population’s health. More and more drinking water studies have applied health risk assessments in their studies [4,5,6,7,8,9,10]. The levels of heavy metals are frequently detectable in various types of drinking water, and these levels can be used to estimate potential health risk. However, these monitoring studies for water contaminants are often accompanied by surveys relying solely on the concentration of heavy metal in drinking water; thus, they fail to account for specific water sources (hand-pressed well water, bottled water, and tap water) and water use habits. Exposure assessment for water contaminants has typically relied upon drinking water consumption patterns rather than water chemical analysis [11].

China is one of the largest producers and consumers of lead and zinc in the world. Chenzhou City has huge reserves of mineral resources and raw material resources and is rich in varieties. It is called the “Nonferrous Metal Village” and it is the most important area of the multi-mineral belt in Hunan Province. In 1985, the collapse of the tailing dam in the Chenzhou lead/zinc mine led to the spread of mining waste spills on farmland along the Dong River. In that disaster, a strip of farmland about 400 m wide on both sides of the Dong River channel was covered with a layer of black sludge that was about 15 cm thick. After the collapse of the dam, an emergency soil cleanup procedure was quickly carried out in some places. The toxic sludge and a major portion of the contaminated surface soil were mechanically removed. Nevertheless, numerous reports indicate that water, soil, vegetables and dust have been heavily polluted by Pb, As, Zn and Cd near the mining areas [12,13,14,15,16].

Exposure to heavy metals via drinking water remains a present health threat to many populations, particularly in areas of developing countries that have insufficient water treatment facilities [17,18,19]. In recent years, numerous health-related pollution incidents associated with heavy metals have been widely reported in China [20,21], especially the high lead levels in the blood of children in Chenzhou [22,23]. These incidents have raised great concern. Previous research showed that the risk perception of drinking water is an important factor on the use of alternative water sources [24]. It is therefore anticipated that the choices of drinking water source in this area might be affected from those reports. However, no survey has yet been conducted in any Chinese region where lead/zinc and lead mineral resources are widely distributed [25], and a large population is potentially exposed to relatively high contaminant levels.

With the significant social and economic achievements that have taken place in the past three decades, lifestyle and health beliefs have drastically changed in recent years in China. Bottled water, barreled water, and household filtrated water have successively entered into family life [26]. More than a single source of potable water is used in each family. Studies have suggested that the choice of a household drinking water source is influenced by many factors [24,27,28], such as socioeconomic status, health risk perceptions, water suppliers, the perception of water quality, trust in water suppliers, familiarity, cultural differences, *etc.* However, water surveys have traditionally been used in epidemiologic, marketing, or economic studies; few studies have incorporated water source and use habit surveys into health risk assessments for heavy metal contamination in drinking water in a rural populations living near a lead/zinc mine.

This study aimed to (1) describe the change in rural residents’ drinking water consumption under the background of the environment; (2) evaluate and compare the risk of human exposure to heavy metals via different drinking water sources; and (3) explore influential factors on household drinking water consumption.

## 2. Material and Methods

### 2.1. Study Area

**Figure 1:**
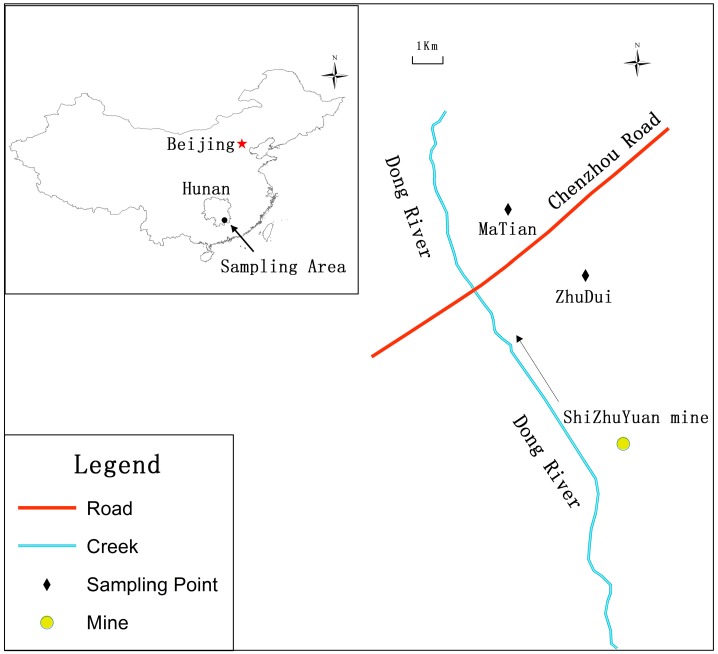
Map of the locations of the two sampling villages in Chenzhou Pb/Zn mine area in Hunan province (southern China).

The study area included MaTian and ZhuDui villages, selected according to previous research [12], in the Suxian District of Chenzhou City, Hunan Province, Southern China. Chenzhou City lies between 24°53′ and 26°50′ N latitude and between 112°13′ and 114°14′ E longitude (Figure 1). The total area of the city is 19,400 km^2^, out of which 241,560 ha are paddy areas and 59,420 ha are vegetable areas. The climate is subtropical, and the average rainfall is about 1500 mm. In the city, about 10,000 people are engaged in mining [13]. The research area is located about 10 km east of the Shi Zhu Yuan mine, which is one of the biggest industrial districts in Chenzhou, and is home to the mining and smelting of Pb, Zn, W, and Mo. In this area, mining activities for heavy metals have been conducted for 500 years. More than 3800 workers are engaged in the mining, smelting, and transport services associated with the mining activities, which continue at full speed today. On 25 August 1985, the big tailing pool dam of the Shi Zhu Yuan mine collapsed because of heavy rainfall [12]. After the accident, some emergency soil cleanup measures were quickly carried out in some places, and a major portion of the contaminated surface soil was mechanically removed. Nevertheless, most of the contaminated farmlands are still presently cultivated. There were some previous studies on heavy metal pollution in soils and plants around this area [12,13,14].

Drinking water in the valley mainly stems from privately dug wells and, to a smaller extent, from the communal water supply of Chenzhou City.

### 2.2. Questionnaire Survey

This was a cross sectional study conducted in Chenzhou, Southern China during August 2013. Firstly, a sampling frame of all households containing at least one qualified subject, aged 18 years or above, living in the study area for more than 1 year, and giving informed consent, was prepared. Those individuals with mental or physical challenges making it too difficult to participate in the study were excluded from the study. A household list was obtained from the township hospitals. Secondly, the two villages are divided into 12 groups according to location and administrative district division; 16 households were selected by simple random technique from each group. One hundred and ninety two households were selected using stratified random sampling method. Efforts were made to interview the head of the household. If the household was locked or no eligible participant was found at the time of interview, the household was either revisited on the subsequent day or an additional household was chosen. All interviews were conducted during the daytime in participants’ homes.

A face-to-face survey of 192 householders in MaTian and ZhuDui village was conducted. The questionnaire was designed to include both socio-demographic characteristic variables and their water consumption. The main research contents are given as follows: (1) contact information, sampling, global positioning system (GPS), re-contact information; (2) socio-demographic characteristics; and (3) water source types, well depths, whether the well adequately sealed, and water treatment facilities. The study was approved by the ethical committee of Xiangnan University. Written informed consent was obtained from every respondent prior to participation. Questionnaires were performed by rigorously trained third-year students of Xiangnan University. The questionnaire was pre-tested with a convenient sample of individuals. Questions that were unclear or otherwise problematic were revised. Subsequently, we performed a pilot study using twenty residents who were randomly selected from our sampling frame. Based on the results, minor revisions were made to decrease the length of the questionnaire. In order to maximize our response rate, health clinicians in the township hospitals and village clinics and the local Center for Disease Control and Prevention went from door to door to mobilize residents to take part in the investigation. A small gift valued at $4 was given to participants to thank them for participation. The response rate for participating in the questionnaire was 100%.

### 2.3. Water Sampling and Determination the Concentrations of Heavy Metals

The water quality survey points were randomly selected proportional to the number of users. According to the research reported by Chinese Center for Disease Control and Prevention [29], 74.87% of rural population use underground water (hand-pressed wells water), and 25.13% use surface water (open wells water and spring water). Moreover, there is only one spring water sample point in the two villages. Therefore, about 12 hand-pressed wells water samples, 2 open wells water samples and 1 spring water sample were selected, respectively. Tap water and barreled water were safe water because their qualities meet the Chinese drinking water standards (GB5749-2006); the concentrations of all the heavy metals were very low. Thus, only one tap water and one barreled water was sampled, respectively. A total of 17 water samples were collected from the two villages.

Water samples were collected on 15 August 2013. Samples were acidified *in situ* with concentrated nitric acid (HNO_3_) to pH < 2 and stored in a refrigerator at 4 °C for further pretreatment. Water sample collection and preservation and the five heavy metals were measured according to standard methods [30]. The concentrations of As and Hg in the water samples were measured with an atomic fluorescence spectrophotometer (AFS-830, Beijing Titan Instruments Co, China), and Pb, Cd, and Zn concentrations were determined by an atomic absorption spectrophotometer (AAS, ZEEnit 700, Analytik Jena, Germany). The detection limits were 0.01, 0.008, 0.008, 0.008 and 8 μg·L^−1^ for Pb, Cd, As, Hg, and Zn, respectively. The coefficient of variation of intra-assay and inter-assay was less than 5%. The recoveries for the six elements were in the range of 94% to 105%.

### 2.4. Health Risk Assessment

Risk assessment normally includes data collection and analysis, exposure assessment, toxicity assessment, and risk characterization.

(a) Exposure Assessment: The exposure dosage through ingestion pathway was calculated by Equation (1) [30]:

(1) $$ADD=\frac{C_{S}\times IR\times EF\times ED}{BW\times AT}$$

where the average daily dose (ADD) is the average daily dose of each metal through ingestion pathway (mg·kg^−1^·day^−1^), Cs the concentration of heavy metal in water (mg·L^−1^), IR is the daily intake of water (L·day^−1^), EF is the exposure frequency (days·year^−1^), ED is the exposure duration(years), BW is the bodyweight (kg), and AT is the average time in days (for non-carcinogens, AT = ED × 365 days; for carcinogens, AT = 70 (human life expectancy) × 365 days).

Due to sampling in the wet season and to avoid the risk heavy metal exposure, the highest concentration of heavy metal in water was used in the calculation. BW (53.6 kg) and IR (1.85 L) used were site-specifically measured and derived from our questionnaire survey.

(b) Risk Assessment: According to the categories of the International Agency for Research on Cancer (IARC), heavy metals are grouped by their potential carcinogenic risks. The carcinogenic and non-carcinogenic risks are usually assessed according to the guidelines in the Risk Assessment Guidance for Superfund of the US Environmental Protection Agency (EPA).

(c) Non-Carcinogenic Risk Assessment: Potential non-carcinogenic risks, as indicated by the hazard quotient (HQ), were evaluated by comparing the exposure dose of chemical contaminants according to each exposure route (water ingestion and dermal absorption) with the corresponding reference dose (RfD) using Equation (2).

(2) $$HQ=\frac{ADD}{RfD}$$

where RfD is the reference dose of heavy metals in a given condition (μg·kg^−1^·day^−1^). The oral reference doses (mg·kg^−1^·day^−1^) were obtained from the US EPA’s Integrated Risk Information System.

(d) Carcinogenic Risk Assessment: The carcinogenic risk of chemical contaminants is usually expressed by a carcinogenic risk factor, CR. When CDI × SF < 0.01, Equation (3) is applicable; otherwise, Equation (4) is used instead.

(3) $$Risk_{i}=ADD\times SF$$

(4) $$Risk_{i}=1-\exp\left(-ADD\times SF\right)$$

where SF is the carcinogenic slope factor (μg·kg^−1^·day^−1^). The calculated value of CR is the cancer-developing probability of any type of carcinogenic chemicals over a life time exposure for a general population. The slope factor and reference dose were obtained from previous study [7,9,31,32]. According to the USEPA’s guidance for acceptable or tolerable carcinogenic risks, the range of the CR value is from 10^−6^ to 10^−4^. In general, if CR < 10^−6^, cancer risks are considered to be negligible; however, if CR > 10^−4^, cancer risks are considered as unacceptable by most international regulatory agencies.

### 2.5. Statistical Analysis

Descriptive and inferential statistics were undertaken to analyze the data. A Chi-square test was performed to evaluate the association between socio-demographic characteristics and using barreled water for cooking. When the expected frequency was less than 5, Fisher’s exact test was used instead. Heavy metal concentrations in different water sources, in different depth and different village had been compared by analysis of variance.

The relation between socio-demographic characteristics and safer drinking water consumption was assessed using binary logistic regression. Tap water and barreled water were generally considered safer because this water was high-quality treated meeting the drinking water standards set up by the Health Department of the Chinese Government. Thus, safe drinking water was defined as barreled water or tap water or using both barreled water and tap water for drinking in the study. A backward stepwise logistic regression model was used. A level of two-sided *p* < 0.05 was considered to be statistically significant. All analysis was performed using SPSS version 13.0.

## 3. Results

### 3.1. Socio-Demographic Characteristics of the Household

A total of 192 households were enrolled in the study conducted in July 2013, in MaTian and ZhuDui village in Chenzhou City, Hunan Province, Southern China. A majority of the heads of household were males (85.4%, *n* = 164). In total, 95.8% (*n* = 184) of the families were living at their current residence. A majority of the families lived in nuclear families with a family size of 3 to 7. Approximately 3% (*n* = 64) of families had family members who had gone out as migrant workers, and 60.9% of participants self-reported that their household income was at the local average. In total, 52.6% of participants reported that their household income was mainly used for food. The percentages of household fuel source supply depended upon were 42.2% on calor gas, 42.7% on coal, 25.5% on wood, and 16.7% on electricity. The vast majority of families (97.9%, *n* = 188) attended the new rural cooperative medical system. Details can be seen in Table 1.

**Table 1:** Socio-demographic characteristics of the study household.

Characteristic	(*N* = 192)	No. (%)
Head of household’s gender		
Male	164	85.4
Female	28	14.6
Lived at current residence		
Always living in	184	95.8
Moved into this place	8	4.2
Family size		
1–2	22	11.5
3–4	87	45.3
5–6	65	33.9
≥7	18	9.4
Family members go out as migrant workers		
Yes	64	33.3
No	128	66.7
Self-reported household income		
Better	29	15.1
Average level	117	60.9
Poor	27	14.1
Poorer	19	9.9
Household income is mainly used for food		
Yes	101	52.6
No	91	47.4
Food supply		
Household production	48	25.0
Half household production half purchasing	75	39.1
Purchasing food at local markets	69	35.9
Fuel type		
Calor gas	81	42.2
Coal	82	42.7
		
Wood	49	25.5
Electricity	32	16.7
Participation in new rural cooperative medical treatment		
Yes	188	97.9
No	4	2.1

### 3.2. Household Drinking Water and Cooking Water Consumption

**Figure 2:**
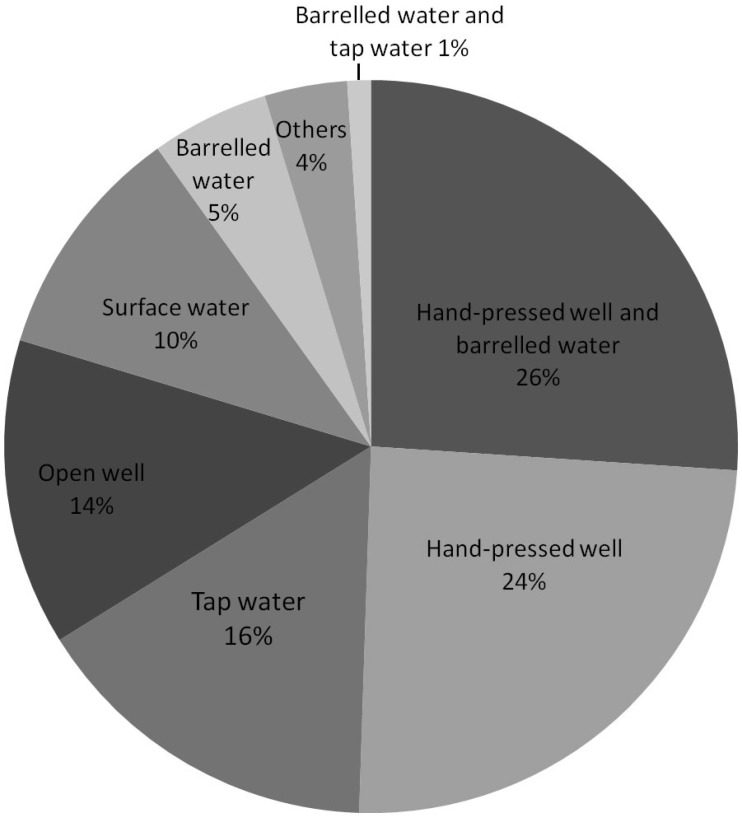
A pie graph of household drinking water consumption.

Results showed that more than half of the households (52.6%, *n* = 101) used hand-pressed well water for drinking; 34.9% (*n* = 67) used barreled water; 17.7% (*n* = 34) used tap water; 15.6% (*n* = 30) used open wells; and 12.5% (*n* = 24) used rivers, streams, ponds, and other surface water. The constituent ratio of household drinking water consumption was found to be as follows: 5.2% (*n* = 10) using only barreled water; 15.6% (*n* = 30) using only tap water; 1.0% (*n* = 2) using both barreled water and tap water; 24.5% (*n* = 47) using only hand-pressed well water; 26.0% (*n* = 50) using both hand-pressed wells and barreled water; 13.5% (*n* = 26) using only open wells; 10.4% (*n* = 20) using only rivers, streams, ponds and other surface water; and 3.7% (*n* = 7) using other combinations (see Figure 2). In 4.2% (*n* = 8) of the households, barreled water was used for cooking. The majority of water consumption of barreled water resulted from the good economic conditions of the households. All cooking use barreled water was reported to be used in the households that self-reported their household income to be at the local average or better. Even so, 11.9% (*n* = 8) of the households using barreled water had “poor” or “poorer” household incomes. Details can be seen in Table 2.

**Table 2:** Household water consumption in the different self-reported household income.

Self-Reported Household Income	Drinking Water				Cooking Use Barreled Water
	Barreled Water and Others	Only Barreled Water	Only Barreled Water and Hand-Pressed Wells water	Only Barreled Water and Tap Water	
Better	18 (26.9%)	5 (50.0%)	11 (22.0%)	0 (0.0%)	3 (37.5%)
Average level	41 (61.2%)	5 (50.0%)	31 (62.0%)	2 (100.0%)	5 (62.5%)
Poor or Poorer	8 (11.9%)	0 (0.0%)	8 (16.0%)	0 (0.0%)	0 (0.0%)
Total	67 (100.0%)	10 (100.0%)	50 (100.0%)	2 (100.0%)	8 (100.0%)

### 3.3. Heavy Metals in Drinking Water Sources

With the exception of Pb, which was higher than the national standard in a hand-pressed wells water sample, the concentrations of heavy metals in drinking water from the two studied communities were well below the Chinese drinking water standards (GB 5749-2006) [29]. The concentration of Arsenic (As) in MaTian is significantly higher than in ZhuDui (*t* = 9.05, *p* < 0.05). The concentration of the five heavy metals in different water sources in different depth was not statistically significantly different. Details can be seen in Table 3.

**Table 3:** The concentrations of heavy metals in drinking water in different villages and different water source.

Category	*n*	Concentration (Mean (Range))				
		Pb (ug·L^−1^)	Cd (ug·L^−1^)	Zn (mg·L^−1^)	As (ug·L^−1^)	Hg (ug·L^−1^)
Water source						
Open wells	2	0.06 (BDL–0.18)	0.029 (BDL–0.054)	0.115 (0.018–0.306)	0.568 (0.034–1.247)	0.081 (0.048–0.13)
Hand-pressed wells						
Depth < 3m	4	3.51 (BDL–10.53 *)	0.227 (BDL–0.53)	0.026 (0.004–0.056)	3.209 (0.262–4.683)	0.070 (0.060–0.076)
Depth = 3~6 m	4	BDL	0.02 (BDL–0.076)	0.027 (0.022–0.039)	1.824 (BDL–5.802)	0.074 (0.030–0.106)
Depth > 6 m	4	BDL	0.126 (BDL–0.35)	0.0195 (0.006–0.033)	2.582 (0.059–6.428)	0.047 (BDL–0.106)
Spring water	1	BDL	BDL	0.027	0.000	0.051
Tap water	1	BDL	BDL	0.025	BDL	BDL
Barreled water	1	BDL	BDL	0.030	BDL	BDL
Villages						
MaTian	9	0.824(BDL~10.53 *)	0.098(BDL–0.53)	0.045 (0.004–0.306)	2.227 (0.034–6.428) **	0.069 (BDL–0.13)
ZhuDui	6	BDL	0.0860(BDL–0.18)	0.026 (0.02–0.03)	0.316 (BDL–0.947)	0.0520
Chinese drinking water standard (GB5749-2006)		10 (ug·L^−1^)	5 (ug·L^−1^)	1(mg·L^−1^)	50 (ug·L^−1^)	1 (ug·L^−1^)

BDL represents below detection limit, The detection limits were 0.01, 0.008, 0.008, 0.008 and 8 μg·L^−1^ for Pb, Cd, As, Hg, and Zn, respectively. * Exceeded the Chinese drinking water standards. ** The concentration of Arsenic (As) in MaTian is significantly higher than in ZhuDui. The concentrations of the five heavy metals in different water sources in differents depth were not statistically significantly different.

### 3.4. The Health Risk Assessment of Heavy Metals in Drinking Water Sources

It is notable that the values provided below are lifetime health risks (with 70 years as the average life span). The risk of human exposure to heavy metals via drinking water are presented in (Table 4). The results showed that the total health risk is 5.20 × 10^−9^~3.62 × 10^−5^, the carcinogenic risk is 5.20 × 10^−9^~3.62 × 10^−5^, and the non-carcinogenic risk is 1.37 × 10^−12^~4.51 × 10^−9^. Carcinogens risk accounted for 99.9% of the total risk. The total health risk were lower than the maximum allowance levels recommended by International Commission on Radiological Protection (ICRP) (5.0 × 10^−5^). The high values of cancer risks associated with exposure to carcinogenic metals via drinking water consumption were estimated to be 3.46 × 10^−5^ for As and 1.59 × 10^−6^ for Cd, while the values of non-cancer risks induced by non-carcinogenic metals were estimated to be 3.71 × 10^−9^, 0.38 × 10^−9^, and 0.13 × 10^−12^ for Pb, Hg, and Zn, respectively. The health risks caused by carcinogenic heavy metals in water were decreased in the following order: As > Cd. The risks by the non-carcinogenic heavy metals decreased in the following order: Pb > Hg > Zn.

**Table 4:** The health risk evaluation of heavy metals via drinking water consumption in two studied villages.

Type	Carcinogenic Risk		Non-Carcinogenic Risk			The Total Health Risk
	Cd	As	Pb	Hg	Zn	
Tap water	1.50 × 10^−9^	3.70 × 10^−9^	0. 88 × 10^−12^	0.49 × 10^−12^	0.41 × 10^−13^	5.20 × 10^−9^
Barreled water	1.50 × 10^−9^	3.70 × 10^−9^	0. 88 × 10^−12^	0.49 × 10^−12^	0.49 × 10^−13^	5.20 × 10^−9^
Open wells	0.16 × 10^−6^	0.92 × 10^−5^	0.06 × 10^−9^	0.32 × 10^−9^	0. 11 × 10^−12^	0.94 × 10^−5^
Hand-pressed wells	1.59 × 10^−6^	3.46 × 10^−5^	3.71 × 10^−9^	0.38 × 10^−9^	0. 13 × 10^−12^	3.62 × 10^−5^
Spring water	1.50 × 10^−9^	3.70 × 10^−9^	0. 88 × 10^−12^	0.25 × 10^−9^	0.84 × 10^−13^	5.20 × 10^−9^

### 3.5. Risk Comparison in Different Drinking Water Sources

With the concentrations of heavy metals in tap water set to 1, we calculated the corresponding ratio of other drinking water in highest value. The total risk of the groups using hand-pressed well, and open well water was significantly higher than the risks for those using tap water, barreled water, and spring water. The total risk for those using hand-pressed well water was 6961 times higher than the risk for those using tap water. It is notable that the values provided below are lifetime health risks (with 70 years as the estimated life span). The total risk of using open well water was 1807 times higher than that using tap water. The total risk of using spring water and using barreled water was the same as tap water. The non-cancer risk of using spring water was 512 times higher than the non-cancer risk of using tap water.

### 3.6. The Influential Factors of Safer Drinking Water Sources

Tap water and barreled water were generally considered safer because this water was high-quality treated meeting the drinking water standards set up by the Health Department of the Chinese Government. So safe drinking water was defined as barreled water or tap water or using both barreled water and tap water for drinking in the study. Table 5 summarizes the associations between safe drinking water consumption and the socio-demographic characteristics of the households. Household drinking water consumption was influenced by family size, household income level, the amount of household income used for food, and food supply sources; conversely, the gender of the head of household, the years at the current residence, the number of family members gone out as migrant workers, and the attendance to the new rural cooperative medical system showed no correlation to drinking water choices. According to a Chi-square test, no variables were significantly associated with the use of barreled for cooking. Those who reported “better household income”, “purchasing food at local markets” and “household income is mainly used for food” were more likely to use safe drinking water; however, those with a family size of seven or more were less likely to use safe drinking water.

**Table 5:** Logistic regression models analysis of socio-demographic characteristics in household safer water consumption.

Characteristics		β	S.E	P	OR (95%CI)
Family size	3–4 (Reference)				
	1–2	−1.899	0.642	0.003 **	0.150 (0.043, 0.527)
	5–6	−0.255	0.360	0.479	0.775 (0.382, 1.571)
	≥7	−0.671	0.623	0.282	0.511 (0.151, 1.734)
Self-reported income level	Average level (Reference)				
	Poor or poorer	0.298	0.446	0.503	1.348 (0.562, 3.231)
	Better	1.551	0.528	0.003 **	4.714 (1.674, 13.279)
Household income mainly using for food	No (Reference)				
	Yes	0.711	0.363	0.050 **	2.036 (1.000, 4.144)
Food supply	Household production (Reference)				
	Half household production half purchasing	0.298	0.446	0.503	1.348 (0.562, 3.231)
	Purchasing food at local markets	1.551	0.528	0.003 **	4.714 (1.674, 13.279)
Participation in new rural cooperative medical treatment	No (Reference)				
	Yes	−2.492	1.276	0.051	0.083 (0.007, 1.009)

Safe drinking water choice was defined as using only barreled water, using only tap water, using only both barreled water and tap water for drinking. ** Significant at *p* < 0.05.

## 4. Discussion

### 4.1. Household Drinking Water and Cooking Water Consumption

Our results show that domestic drinking water choices noticeably changed. A prior survey reported that, in China, the main source of rural drinking water in water was mainly underground water, with 74.87% of the studied rural population using underground water sources, and 25.13% using surface water. In total, 55.10% of the rural population used water from centralized water supply systems. The use of domestic barreled water increased and the use of hand-pressed well water decreased, compared to the water uses of other rural residents [33]. In our study, 52.60% used hand-pressed well water for drinking, 24.48% of households used only hand-pressed well water, and 26.04% used both hand-pressed well and barreled water. In total, 5.21% of households used only barreled water for drinking, and 4.17% of the households even used barreled water for cooking. This suggests that tap water was not the main source of drinking water, hand-pressed well water was predominantly used, but barreled water was popularly used by local people for drinking and cooking. The reason might be related to people’s risk perceptions of heavy metals. When people feel there are risk to their health as a result of a variety of media sources, adaptive behavior (changing the choice of drinking water sources) will be occur to avoid risk [27,34,35].

### 4.2. Water Quality Assessment and Health Risk Assessment of Heavy Metals

In order to investigate the drinking water quality and evaluate the risk of human exposure to heavy metals via drinking water sources, fifteen samples collected from the household were analyzed and concentrations of five heavy metals were measured in drinking water. We found that, besides Pb, in a hand-pressed well water sample, the other heavy metal contents were lower than the national standards. In total, 6.67% exceeded the Chinese drinking water standards. The findings supported previous similar findings. Previous studies showed that in their study areas, the water quality in poor, good, and excellent status during the dry season accounted for 5%, 10%, and 85% of the total rural ground water sources, respectively, while during the wet season, it accounted for 5%, 5%, and 90%, respectively. This was determined using a single-factor assessment method and Nemerow index methods at twenty sampling points [36]. The measurements from fifteen well water samples indicated that the collapse of the tailing dam and mining activity in the area does not distinctly impair the natural quality of most of the well water.

Based on the concentration of heavy metals in drinking water sources, the carcinogenic and non-carcinogenic risk assessments were performed according to the methods recommended by the United States EPA. Our study found that the total health risk of five metals was lower than the maximum allowance levels recommended by ICRP (5.0 × 10^−5^). The results indicate that the health risk for the five heavy metals in the region were at acceptable levels for drinking water sources. Perhaps, the local people’s perception of the risk of heavy metal exposure via drinking water was actually overestimated. Too much worry about the risks of drinking water is unnecessary.

The Pb was in excess of the standards, but its health risk was lower than those of arsenic and cadmium. Carcinogenic risks accounted for 99.9% of the total risk. This indicated that the health risk caused by the carcinogenic heavy metals was much larger than those caused by the non-carcinogenic heavy metals, and As was the greatest potential risk. Corresponding pollution control strategies may need to be implemented for public health protection purposes. In the non-carcinogenic pollutants, Pb is the priority controlled pollutant in this region.

In order to evaluate the effectiveness of people’s behaviors, we compared the health risk in different drinking water sources. With the concentrations of heavy metals in tap water set at 1, we found that the total risk of using hand-pressed well and open well water group was significantly higher than tap water, barreled water, and spring water. The total risk of using hand-pressed well water was 6961 times higher than that of using tap water. The total risk of using open well water was 1807 times higher than that of using tap water. The total risk of using spring water and using barreled water was the same as that of tap water. The non-cancer risks of using spring water were 512 times higher than that of using tap water. It is notable that values provided below are lifetime health risks (70 years as the life span). The survey shows that different choices of drinking water source led to different health risks. Using safe drinking water can remarkably reduce the risk of the ingestion of heavy metals through water. These results coincide with those of previous studies [4,37,38].

### 4.3. The Influential Factors of the Choice of Drinking Water Sources

In order to provide references for later work in the field of environmental health education and behavior intervention, the relation between safer water choices and socio-demographic characteristics was firstly assessed by univariable logistic regression. Our findings show that safer household water choices were correlated with household income level, family size, the household income being mainly used for food, and food supply source. These findings are in line with previous studies [26,39,40]. A strong correlation was found between household income level and domestic drinking water choices (*p* = 0.003) using a backward stepwise logistic regression model. Out of the three household income level groups, the better household income group was about five times (odds ratio 4.71) more likely to use tap water and barreled water than the average level group. However, the poor and poorer income level groups were less significant (*p* > 0.05) than the average level group. Family size, household income mainly being used for food, and the choice of food supply source were also found to be closely related to household income level. The greater the family size was, the more spending was found to be used for drinking water. Perhaps, in order to save money, some families tended to choose hand-pressed well water because it does not require additional money.

There are several limitations to the current study. This study only measured five kinds of heavy metals in drinking water to evaluate the risk of harm to human body health. Our approach only considered the intake levels during the wet season, without considering the other poisonous and harmful substances that can produce health risks; therefore, the evaluation actually underestimated the risk of heavy metal exposure. Daily water consumption, exposure frequency, exposure duration, and people’s risk perceptions of heavy metals were not included in this investigation. Selection bias and underrepresentation may have occurred because only two village residents were recruited and 17 water samples were selected for detection. Thus, in this paper, the drinking water health risk assessment of heavy metals in the region is preliminary.

## 5. Conclusions

We found that hand-pressed well water was mainstream, while the use of drinking water sources noticeably changed with an increase in barreled water. With the exception of Pb, the concentrations of heavy metals in drinking water were well below the Chinese drinking water standards. The total health risk caused by the five metals was lower than the maximum allowance levels recommended by the ICRP and was at acceptable levels for drinking water sources. As and Pb are priority controlled pollutants in this region. The total risk of using hand-pressed well water in highest value was 6961 times higher than that of using tap water. Using safe drinking water (tap water and barreled water) can greatly reduce the risks of ingesting heavy metals through drinking water. Households making safer water choices were correlated with household income level, family size, the household income mainly being used for food, and food supply source. A strong correlation was also found between household income level and household drinking water choices.
